# Low Complexity Regions in Mammalian Proteins are Associated with Low Protein Abundance and High Transcript Abundance

**DOI:** 10.1093/molbev/msac087

**Published:** 2022-04-28

**Authors:** Zachery W. Dickson, G. Brian Golding

**Affiliations:** Department of Biology, McMaster University, Hamilton, ON, Canada

**Keywords:** low complexity, protein abundance, transcript abundance, protein regulation

## Abstract

Low Complexity Regions (LCRs) are present in a surprisingly large number of eukaryotic proteins. These highly repetitive and compositionally biased sequences are often structurally disordered, bind promiscuously, and evolve rapidly. Frequently studied in terms of evolutionary dynamics, little is known about how LCRs affect the expression of the proteins which contain them. It would be expected that rapidly evolving LCRs are unlikely to be tolerated in strongly conserved, highly abundant proteins, leading to lower overall abundance in proteins which contain LCRs. To test this hypothesis and examine the associations of protein abundance and transcript abundance with the presence of LCRs, we have integrated high-throughput data from across mammals. We have found that LCRs are indeed associated with reduced protein abundance, but are also associated with elevated transcript abundance. These associations are qualitatively consistent across 12 human tissues and nine mammalian species. The differential impacts of LCRs on abundance at the protein and transcript level are not explained by differences in either protein degradation rates or the inefficiency of translation for LCR containing proteins. We suggest that rapidly evolving LCRs are a source of selective pressure on the regulatory mechanisms which maintain steady-state protein abundance levels.

## Introduction

Low Complexity Regions (LCRs) are some of the most common shared motifs in eukaryotic proteins (Golding, 1999; Huntley and Golding, 2000). These regions are highly repetitive and are enriched for one or a few amino acid residues. These regions are often intrinsically disordered, lacking fixed structures under normal physiological conditions (Romero et al., 2001). Perhaps as a result, these regions were thought of as a protein analog for “junk-DNA,” or as spacers between other protein regions (Golding, 1999). However, more research has shown that these regions can perform various specific roles. They have been associated with phenotypic variation (Fondon and Garner, 2004), implicated in neurodegenerative diseases (Cummings and Zoghbi, 2000), suggested as hub proteins for interaction networks (Dosztanyi et al., 2006), and shown to be essential to the normal functioning of some proteins (Loya et al., 2012).

LCRs are broadly defined by compositional bias (Mier et al., 2019), and there exist multiple methods for detecting and classifying LCRs. The basis for these methods range from sequence entropy (Wootton and Federhen, 1993) and probability (Harrison, 2017) to prediction of intrinsic disorder (Dosztányi et al., 2005). LCRs can be classified in several different manners including: by primary amino acid, by location in the protein, by length, and by function (if known). A recent study found that for some proteins containing an essential LCR, the region could be replaced with some LCRs from other proteins without loss of function (Loya et al., 2017). The inter-operability of LCRs could thus be used as another classifier.

LCRs can expand and contract rapidly via slippage of DNA polymerase during replication (Huntley and Golding, 2006), and can arise from unequal crossover events (DePristo et al., 2006). They may also evolve as the result of selection. Whether the LCR is retained in the protein once it has arisen is affected by several factors. Recent work has suggested that LCRs are preferentially retained in proteins which are already tightly regulated, possibly as the existing regulation ameliorates any deleterious effects from the LCR’s presence (Chavali et al., 2017).

LCRs are also thought to arise in regions under relaxed selection, however previous work, examining serine homopolymers, found evidence of selection based on codon usage (Huntley and Golding, 2006). Lenz et al. (2014) found that substitution rates increase in primate proteins in those regions flanking repetitive sequences like LCRs and microsatellites, and that these regions were under higher purifying selection. All of these results suggest that the presence of LCRs has evolutionary consequences for their host proteins.

It is well known that expression levels are positively correlated with selection pressure, with those genes which are most highly and broadly expressed being under strong selection (Pál et al., 2001). The abundance of these proteins makes their fitness sensitive to perturbations in their function as defined by their structure (whether globular or intrinsically disordered). The appearance, expansion, and deletion of an LCRs all have the capacity to dramatically alter the ability of a protein to perform its function. The majority of such mutations are deleterious and would be subject to purifying selection, and only tolerated where the effect is smaller, such as low abundance proteins under more relaxed selection. The intolerance for LCRs in high abundance proteins would result in a negative association between protein abundance and the presence of LCRs. It would then be expected that LCR-positive (LCR^+^) proteins would be have lower expression than LCR^−^ proteins.

Previously, this relationship has only been incidentally examined. Some specific LCR^+^ proteins have been studied for their influence on human health or their structural properties (Cornman and Willis, 2009; Shin et al., 2016). A more general study of *S. cerevisiae* proteins which contained homo-repeats found that these proteins are in lower abundance than other proteins (Chavali et al., 2017). This study examined only this one type of LCR which may have different properties from other LCR types.

Characterizing the relationship between LCRs, gene expression, and protein abundance (PAb) is an opportunity to shed light on the complex relationship between the latter two. There are multiple levels of regulation applying to protein expression at every step from transcription, through translation, and protein stability. Not all of these processes are well understood and thus attempts to predict PAb from mRNA levels have been met with mixed success (Nie et al., 2006, 2007). This is a concern as gene expression research is increasingly being used to develop therapies, despite weak connections to the more physiologically relevant PAb. Nie et al. (2006) have used sequence characteristics to address a portion of the variation observed. Among the characteristics examined were amino acid and codon usage, but LCRs were not considered.

To our knowledge, the research here is the first to comprehensively examine LCR^+^ protein expression across mammals. We characterize the relationships between LCRs of different types and their expression in various tissues. We examine the apparent differences between transcript abundance (TAb) and PAb through the lens of LCRs and show that PAb is negatively associated with the presence of LCRs, but TAb is, unexpectedly, positively associated with the presence of LCRs.

## Results

**Fig. 1.**
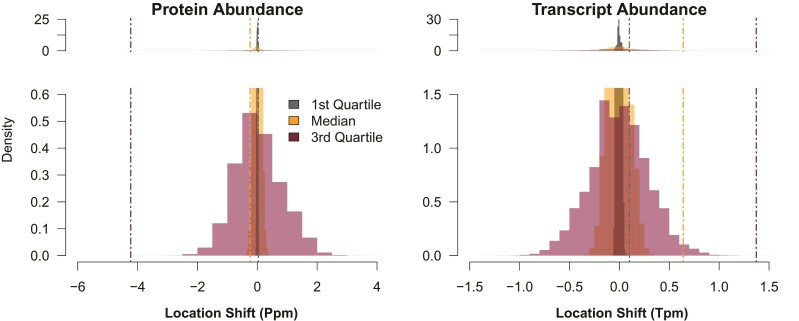
Under permutation all abundance quartiles are significantly different based on LCR status. The distribution of shifts in abundance quartiles after one million permutations is shown; the lower plots are insets of the upper plots. The observed shift for each quartile (dashed lines) can be compared to the matching distribution of location shifts under permutation. LCR^+^ proteins have lower abundance for all quartiles (P<0.023) but higher for TAb at all three quartiles ($P\leq 2\times 10^{-6}$).

Human TAb and PAb from the GTEx project and the PaxDb were collected for 17,975 proteins. Of these, 4,246 (23.6%) were identified as containing an LCR as determined by the presence of a 15 amino acid window with Shannon entropy less than 1.9 bits. One million random permutations of the labeled LCR status were performed to generate distributions of expected quartile shifts. The observed median PAb for LCR^+^ proteins was lower than that found in 99.2% of the permutations. On the other hand, the observed median TAb was higher than all medians found in the permutations (fig. 1). In both cases, the shift is significantly different from zero by the Mann–Whitney *U* test ($P_{\mathrm{PAb}}<10^{-16}$, $P_{\mathrm{TAb}}=1.06\times 10^{-11}$).

**Fig. 2.**
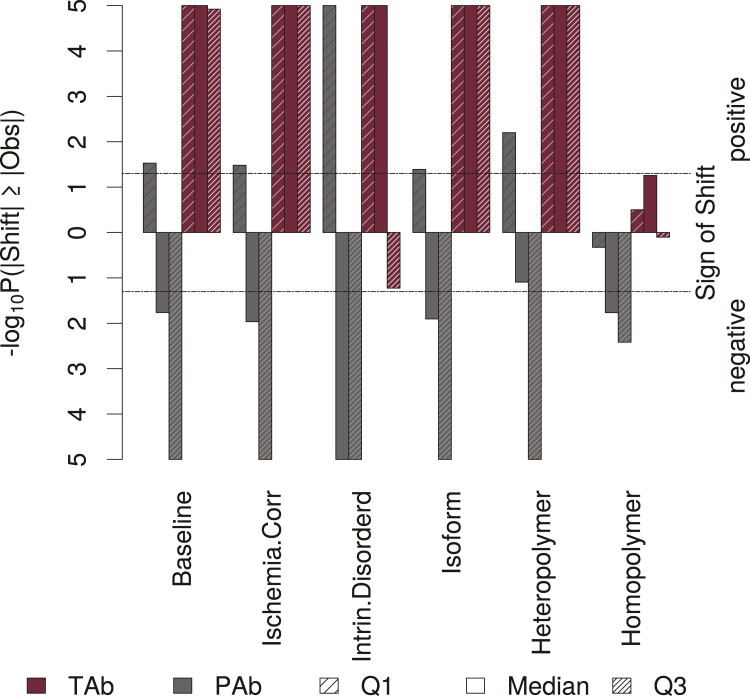
Observed shifts in LCR status remain after controlling for several technical explanations, and known biological conditions. Bars represent empirical *P* values, calculated as the proportion of 100,000 permutations of GTEx and PaxDB human data with quartile shifts at least as large as the observed shift. Bars above the horizontal axis have observed shifts where LCR^+^ proteins are greater than the null expectation, while bars below the horizontal axis represent observed shifts where LCR^+^ proteins are below the null expectation. Dotted horizontal lines indicate a significance threshold of 0.05. All results are qualitatively similar to the baseline analysis.

Figure 2 shows the significance of the observed shift for the baseline, entropy-based permutation and several potential biasing factors. Permutation testing using intrinsic disorder instead of LCR status yielded qualitatively similar results with two exceptions. The bottom quartile of abundance for LCR^+^ proteins shows significantly greater abundance than that for LCR^−^ proteins, and there was no significant difference observed for the top quartile of TAb. Ischemia-time-adjusted TAb values give near identical results to the unadjusted TAb values. Qualitatively similar results are also observed when accounting for isoform redundancy, and restricting to only heteropolymer LCRs. The shifts observed for homopolymer LCRs are much less significant, but are qualitatively similar.

**Fig. 3.**
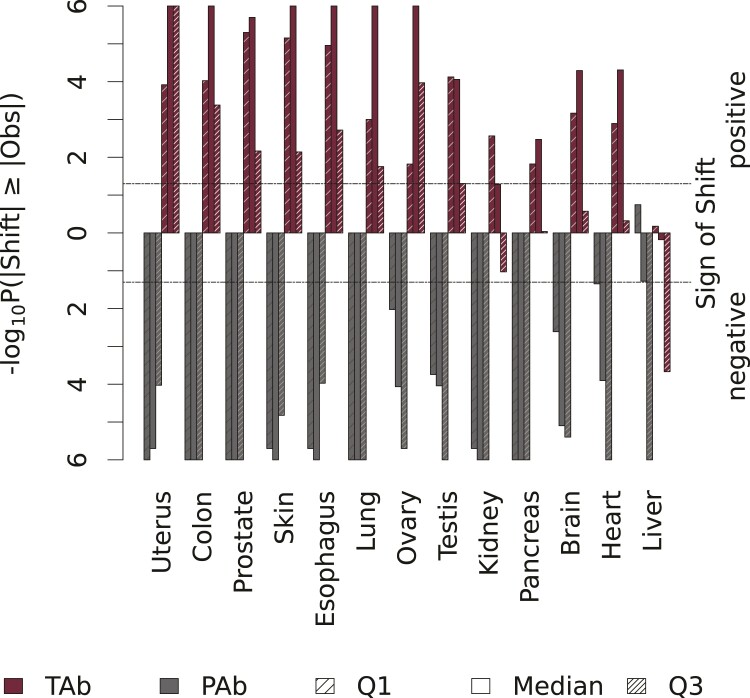
Differences in abundance between LCR^+^ and LCR^−^ proteins are consistent across tissues in humans. Bars represent empirical *P* values, calculated as the proportion of 1×106 permutations of GTEx and PaxDB human data with quartile shifts at least as large as the observed shift. Bars above the horizontal axis have observed shifts where LCR^+^ proteins are greater than the null expectation, while bars below the horizontal axis represent observed shifts where LCR^+^ proteins are below the null expectation. Dotted horizontal lines indicate a significance threshold of 0.05. TAb shifts are consistently, significantly positive while the PAb shifts are consistently, significantly negative.

The median tau index of LCR^+^ proteins (0.74) is lower than that for LCR^−^ proteins (0.76) ($P<10^{-5}$) indicating that LCR^+^ proteins are more common among broadly expressed proteins. As a result the proportion of LCR^+^ proteins is higher than in the aggregate. These proportions vary from 24.1% in the testis to 25.1% in the brain. Regardless of these differences, the aggregate permutation results are qualitatively consistent with the results across tissues (fig. 3). Liver tissue is an exception, however it had the smallest number of expressed proteins (12,914), the second lowest proportion of LCRs among those proteins (24.6%), and the highest standard deviation in $\log_{2}$ scaled TAb (3.15).

These results suggest that the presence of LCRs is associated with an increase in the level of TAb which might be required to maintain a particular PAb level, as compared to LCR^−^ proteins. While there are many processes in the pathway from gene transcription to protein degradation we focussed on the rates of protein degradation and translation. Coefficients of degradation ($k_{\deg}$) for 3,222 human proteins were aggregated, of which 965 (30.0%) were LCR^+^. Schwanhäusser’s data for 2,180 mouse proteins included 450 (20.6%) LCR^+^ proteins. In both datasets, LCR^+^ proteins degrade 20–30% more rapidly (table 1).

**Table 1.** Summary of Protein Degradation and Translation.

Process	Species	LCR Status	*N*	Median	95% CI	
					Lower	Upper
Degradation	Human	−	965	1.82×10−2	1.75×10−2	1.88×10−2
k.deg (1/h)		+	2,257	2.42×10−2	2.28×10−2	2.57×10−2
	Mouse	−	450	1.38×10−2	1.30×10−2	1.45×10−2
		+	1,730	1.57×10−2	1.46×10−2	1.79×10−2
Translation	Human	−	4,259	2.90×10−2	2.84×10−2	2.95×10−2
TWnTE		+	13,795	1.41×10−2	1.37×10−2	1.48×10−2
	Mouse	−	865	2.28×10−4	2.20×10−4	2.37×10−4
		+	2,542	1.47×10−4	1.38×10−4	1.54×10−4

The perturbability and resupply of local codon supply were estimated using Schwanhäusser’s mouse data. The estimated parameters indicated low perturbability but slow resupply meaning translation is only likely to be affected for longer, more repetitive transcripts. A single translation step consumes 5.78% of the tRNA isoacceptor supply of the least supplied codon, while 0.064% of the deficit between local and global supply is ameliorated. These parameters result in a correlation between measured translation rates and calculated Time Weighted normalized Translation Efficiency (TWnTE) values of 0.53 (95% CI [0.51,1.0]).

Using these translation parameters and selective wobble constraints optimized for each dataset (Supplementary table S2, Supplementary Material online), TWnTE values were calculated using GTEX and Schwanhäusser data for 18,054 (30.9% LCR^+^) human and 3,407 (34.0% LCR^+^) mouse proteins. For both species, transcripts encoding LCR^+^ proteins are translated 35–55% less efficiently (table 1).

**Fig. 4.**
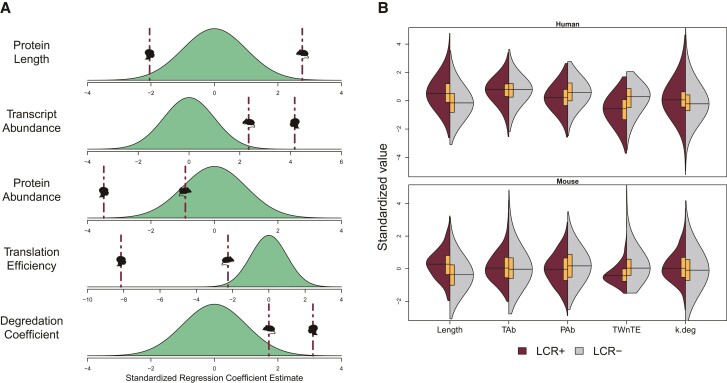
Logistic regression shows PAb and TAb are significantly associated with the probability of a protein containing an LCR. Regression is based on GTEx and PaxDB human abundance data as well as Shwanhäusser mouse data controlling for protein degradation and translation efficiency. Regressors are standardized so that the effect magnitudes may be compared (*A*) Estimated regression coefficients (maroon lines) are compared to a standard normal distribution (Teal). PAb and LCRs are negatively correlated, while the opposite is true for TAb. (*B*) Split violin plots showing the distributions of the regressors’ values across LCR^+^ (maroon) and LCR^−^ (gray) proteins. Yellow bars indicate the median, and interquartile for the distribution in which the bar is embedded.

Logistic regression was used to estimate the relationship between abundance and LCR status while accounting for protein degradation, translation efficiency, and the increased odds of finding LCRs in longer proteins. There were complete data for 3,107 (29.1% LCR^+^) human proteins and 2,155 (20.7% LCR^+^) mouse proteins. Figure 4 shows the standardized regression coefficients. Despite including two regulatory steps and the length of the proteins, PAb is still negatively associated with the presence of LCRs. From the coefficients in Supplementary table S3, Supplementary Material online, we estimate that a human protein which has double the abundance of an otherwise similar protein would have 5.5 (95% CI [2.5,8.5])% lower odds of having an LCR. Conversely TAb is positively associated with the presence of LCRs. A doubling in TAb is associated with an 8.9 (95% CI [4.6,13])% increase in the odds of encoding an LCR. The PAb results are not significant for mice but tend towards a negative relationship with a 2.6 (95% CI [−3.0,7.9])% odds reduction for doubling PAb. The relationship with TAb is qualitatively the same between human and mouse proteins. For mice, the odds of encoding an LCR are 13 (95% CI [2.1,25])% higher for each doubling in TAb. There was no qualitative difference in the relationships to either PAb or TAb with changes to the translation parameters used when calculating TWnTE, or even whether TWnTE was included in the regression (Supplementary fig. S1, Supplementary Material online).

**Fig. 5.**
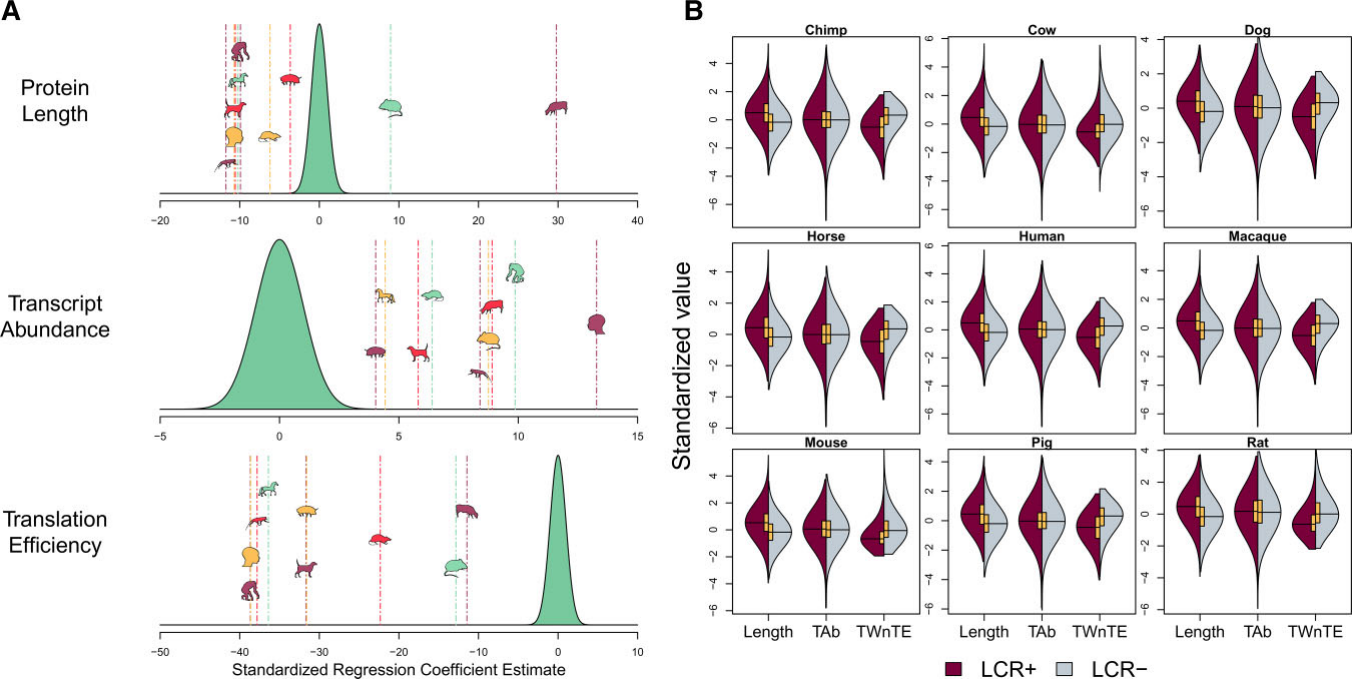
TAb is associated with an increased probability of an LCR being present, based on consistently processed RNA-Seq data from nine mammalian species. Regressors are standardized so that the effect magnitudes may be compared. (*A*) Estimated regression coefficients (contrastingly colored lines) are compared to a standard normal distribution (Teal). In all cases, TAb is positively associated with the presence of LCRs. (*B*) Split violin plots showing the distributions of the regressors’ values across LCR^+^ (maroon) and LCR^−^ (gray) proteins. Yellow bars indicate the median, and interquartile for the distribution in which the bar is embedded.

While PAb and degradation data are not as readily available across mammalian species, RNA-Seq data are plentiful. Therefore, transcriptomic data were processed together and aggregated for nine mammalian species. As raw RNA-Seq results were not available for the GTEx or Schwanhäusser datasets, the human and mouse data are not the same as in the previous analyses. The results of a logistic regression of LCR status against TAb, protein length, and TWnTE can be seen in figure 5. Each regression used data from at least 30 k proteins, with LCR^+^ rates between 23.0% (Human) and 27.6% (Horse). See Supplementary table S3, Supplementary Material online for details. The positive relationship between TAb and the presence of LCRs was qualitatively consistent across mammals. All estimates for the increase in odds of encoding an LCR were between 1.5% and 4.4% for each doubling in TAb.

## Discussion

As would be expected if there were selective pressures against evolutionarily unstable regions in highly abundant proteins, we have found that PAb is negatively associated with LCRs. However, the opposite is true at the level of TAb where LCR encoding transcripts have higher abundance than expected. The observed associations are consistent across mammalian taxa. This is true even when accounting for two of the processes along the pathway from gene expression to protein degradation. This indicates that the associations between LCRs and abundance cannot be explained solely by reduced translation efficiency of repetitive sequences or elevated degradation rates of LCR^+^ proteins.

We investigated several technical explanations of the observed effect, the first of which was whether the effect was an artifact our choice of LCR threshold. We reanalyzed our data with minimum entropy thresholds ranging from 0 to 2.2 bits and observed that elevated TAb for LCR encoding transcripts is only observed for thresholds between 1 and 2. Increasing the entropy threshold dilutes the effect of LCRs via the inclusion of more false-positives in the LCR^+^ category. This reduces the observed differences between the two groups. Conversely, the loss of signal with lower thresholds is due to a loss of statistical power. With the proportion of LCR^+^ proteins dropping from 24% at a threshold of 1.9 down to 4% at a threshold of one, the power to detect an effect as large as we have observed drops below 0.5. Our chosen threshold strikes the balance between sample size, while still limiting the analysis to proteins with minimum entropies which are correlated with biological effects. This is further supported by the qualitatively similar results when looking at intrinsically disordered protein regions which often overlap LCRs (fig. 2).

We also investigated the possibility that bias in the mapping of short reads to highly repetitive sequences would explain the elevated TAb we observed. In that case, it would be expected that within a transcript, the LCR encoding region would have a higher depth of coverage than LCR^−^ regions. As we had access to the raw reads for the mammalian RNA-Seq experiments, we were able to evaluate this and found that there was no significant difference in depth of coverage for LCR^−^ encoding regions.

The data from the GTEx project is generated from human donors and time does pass between the death of the donor and stabilization of tissues for RNA-Seq. This ischemia time may have biased TAb towards more stable transcripts as unstable transcripts would be degraded during the ischemic window. If the observed shift in TAb were the result of this bias it would suggest that LCR encoding transcripts are more stable. However, this is not the case. Almost identical effects are observed when using unadjusted or ischemia-time-adjusted TAb values (fig. 2). This indicates that the observed effect is not the result of a bias towards more stable transcripts.

The GTEx/PAb analysis had a small potential for redundancy as a result of protein isoforms. Of the 18,016 genes for which we obtained complete TAb, PAb, and LCR data, 49 had data from multiple isoforms. The abundance of each isoform was unique, and we observed near identical results to the baseline observations (fig. 2).

Chavali et al. (2017) have previously shown that yeast proteins which contain amino acid homopolymers have lower abundance than homopolymer free proteins. We repeated our permutation analysis twice: comparing only homopolymer containing proteins to LCR^−^ proteins, and comparing only heteropolymer LCRs to LCR^−^ proteins (fig. 2). We found a much weaker signal for homopolymer containing proteins, likely due to a lack of statistical power as only 536 of the 4,259 LCR^+^ proteins had homopolymers. Homopolymer LCRs were qualitatively similar to the baseline results, but do not completely drive the effects we have observed, as they are consistent for heteropolymer LCRs as well.

Data availability presented a limitation to our ability to interrogate the biological mechanisms driving the elevated TAb of LCR encoding transcripts and the prevalence of elevated TAb and reduced PAb across mammals. For the latter, proteome wide PAb data are not widely available across mammals. However, the consistent observation of a positive association at the transcript level across mammals may indicate that the same relationship observed for humans and mice holds across mammals for PAb. Regardless, our work shows that there can be a disconnect between transcript and protein levels. This highlights the importance of carefully investigating RNA-Seq-based conclusions to ensure that the physiologically relevant proteins are likewise up- or down-regulated.

The lack of translation rate data across mammals also limited the confidence in the accuracy of our calculations of TWnTE. However, we demonstrate that the particulars of the calculation did not significantly impact the conclusions about the relationships between TAb, PAb, and the presence of LCRs (Supplementary fig. S1, Supplementary Material online). Regardless, we believe that TWnTE is a useful method for calculating translation efficiency as it goes beyond merely considering sequence composition. In contrast to the standard normalized Translation Efficiency (nTE), TWnTE allows us to account for the inherently ordered nature of the codons in a transcript. This method of calculation clearly shows a difference in translation efficiency between transcripts for LCR^+^ and LCR^−^ proteins as seen in figures 4 and 5.

The inclusion of TWnTE does make the interpretation of coefficients for the logistic regression more difficult. TWnTE, as calculated with a low coefficient of resupply, is highly correlated with protein length; longer proteins have lower TWnTE. This correlation and the differences in TWnTE between the GTEx human and Schwanhäusser mouse data cause the apparent difference in effect for protein length in figure 4. However, the main goal of this analysis was to assess the relationship with TAb and PAb which are not strongly correlated with any of the other parameters. As a result, our conclusion that the presence of an LCRs is positively associated with TAb is unaffected.

Aside from data limitations, our analyses are also limited to an aggregate view across the wide variety of LCR compositions and properties. LCR composition is associated with variation in both transcript and protein abundance as shown by Cascarina and Ross (2018). They observed that PAb, nTE, and protein half-life can have qualitatively different relationships depending on the primary amino acid in an LCR (Cascarina and Ross, 2018). Supplementary figure S2, Supplementary Material online shows the results of logistic regression for human GTEx data when the most prevalent amino acid in the minimum entropy regions of a protein are included as an interaction term with PAb and TAb. For statistically significant coefficient estimates, the observation made in aggregate holds true. PAb is negatively associated with LCRs, and TAb is positively associated with LCRs. Glycine, the least conformationally restricted amino acid, is the sole exception. The positive association of PAb with LCRs in proteins where glycine is the primary amino acid in low entropy regions may be driven by the high frequency of glycine in abundant structural proteins such as keratin (Parry and North, 1998) and collagen (Persikov et al., 2000) which have repeating structures.

The structural function of these LCRs are undoubtedly a subset of the many important functional roles LCRs fulfil. These roles require a particular level of abundance to be maintained, leading to selective pressures on mechanisms which regulate abundance. Our proposed explanation of the disconnect between TAb and PAb for LCR^+^ proteins is that elevated TAb is an adaptive response to the appearance of LCRs in protein sequences. While the processes we investigated did not explain the disconnect, it is likely that through the combined effect on multiple regulatory processes LCRs lead to a reduction in steady-state protein levels. As these proteins still carry out important functions, there is a selective pressure to counter the LCR-associated reduction. Either increased transcription or stabilization of LCR encoding transcripts may be the specific adaptive response leading to elevated TAb. Follow-up studies will examine the ancestral states of the proteins and their abundance to examine this hypothesis. As well as to determine the specific biological mechanism leading to decreased PAb and yet increased TAb of LCR^+^ proteins.

## Materials and Methods

PAb data for human proteins were downloaded from PaxDb v4.1 (Wang et al., 2012). Data from brain, colon, esophagus, heart, kidney, liver, lung, ovary, pancreas, prostate, skin, testis, and uterus tissues were integrated with each protein being assigned abundance equal to the median abundance across tissues in which the protein was expressed. TAb data were downloaded from the GTEx project v8 (GTEx Consortium, 2013). Data for the 13 tissues listed above were integrated in the same way to give a median across tissues where the transcript is expressed. The exclusion of tissues with zero measured expression maximizes the number of proteins which can be used in the analysis as half of transcripts have zero abundance in the majority of the selected tissues in the GTEx data. While there is variance in the abundance of a protein and its transcript across tissues, the sequences comprising LCRs remains constant across tissues.

Breadth of expression and initial expression were calculated from the raw GTEx TAb data. The former was quantified using the tau index (Yanai et al., 2005). This is index ranges from 0 to 1, where 0 indicates a gene expressed in all tissues equally and a 1 indicates a tissue which is expressed only in one tissue. The TAb at time of death for GTEx data was estimated by fitting exponential curves to TAb as a function of ischemia time across samples for each transcript in each tissue.

Transcript and Protein Sequences were downloaded from the Ensembl database, release 99 (Howe et al., 2021). As identifiers used across studies differed, all sequence identifiers were mapped to UniProt protein identifiers using the UniProt Retreive/ID mapping service (The UniProt Consortium, 2017). The 13 mitochondrial encoded proteins with both TAb and PAb data were excluded as mitochondrial genes are under fundamentally different constraints from the majority of nuclear genes.

LCRs in protein sequences were identified using the SEG algorithm (Wootton and Federhen, 1993) using a window of 15 amino acids, a lower complexity bound of 1.9, and a higher complexity bound of 2.5 as these parameters were shown to detect longer, more repetitive regions in previous research (Golding, 1999). This value also represents the lower inflection point in the distribution of minimum entropies across human proteins. The overlapping property of intrinsic disorder was also calculated. Proteins with intrinsically disordered regions were identified using IUPred (Dosztányi et al., 2005) in glob mode. A protein was considered to have an intrinsically disordered region if it contained a nonglobular region.

As LCRs range from homopolymer tracts to compositional bias, we subdivided observed LCRs into homo- and heteropolymer LCRs. An LCR was considered a homopolymer if there was a contiguous tract of a single amino acid which made up at least half the length of the LCR.

Mouse TAb and PAb, as well as protein degradation rates, and translation rates were extracted from data generated by Schwanhäusser et al. (2011). Human Protein degradation rates were integrated from multiple sources (Doherty et al., 2009; Cambridge et al., 2011; Zhang et al., 2016; Zecha et al., 2018) by first converting all reported values to the coefficient of degradation. The geometric mean value of the coefficient across studies for each protein was used.

Translation efficiency was calculated based on the transcript sequences, in a method derived from the nTE scale (Pechmann and Frydman, 2012). On this scale, the translation efficiency of a transcript is the geometric mean of the translation efficiencies for each codon within the transcript. The value for each codon is calculated as the ratio of the supply of tRNA isoacceptors for a codon to the global usage of that codon. This translation efficiency scale does not account for the ordering of codons within a transcript, which can have a profound effect through local tRNA depletion. For this work, the codon usage values are calculated using equation (1) as described by Pechmann and Frydman (2012), however the standard calculation of codon supply is treated as initial conditions for the translation of a transcript. For each subsequent codon in a transcript, the local supply of tRNA isoacceptors is updated according to equation (2): accounting for perturbation of the local supply as well as resupply from the cellular environment. The perturbability is the proportion of the local supply used, scaled to the least supplied codon. Resupply is the portion of the local deficit which is ameliorated at each time step. Calculating TWnTE allows for codons which appear at the end of a repeat to have lower translation efficiency than those which appear alone or at the start of a repeat.

(1) $$S_{i,0}=\sum_{\;j=1}^{n_{i}}(1-s_{i,j})N_{i,j}/\max S_{0}$$

where: $n_{i}$ denotes the number of tRNA isoacceptors for codon *i*; $s_{i,j}$, wobble constraint between codon *i* and the *j*th tRNA; $N_{i,j}$, the copy number of codon *i*’s *j*th tRNA

(2) $$S_{i,t}=\beta S_{i,0}+(1-\beta )(1-\alpha_{i})S_{i,t-1}$$

where $S_{i,0}$ denotes normalized initial codon supply; $S_{i,t}$, local codon supply for the codon *i* at time *t*; $\alpha_{i}$, normalized perturbability for codon *i*; β, Coefficient of resupply of tRNA isoacceptors.

The selective constraints on wobble base pairing are a measure of how tolerant the ribosome is of different types of mismatches between codon–anticodon pairs. Most mismatches are not tolerated, but values were allowed to vary between 0 (tolerant) and 1 (intolerant) for A-A, U-G, G-U, and A-C mismatches. Wobble constraints were set for each organism by optimizing the correlation between codon supply and codon demand with R (R Core Team, 2013) using the neldermead package (Bihorel and Baudin, 2018), with initial conditions from estimates generated for yeast (dos Reis et al., 2004). Codon supply was determined from genomic tRNA counts which can vary widely even in mammals. For example, *Bos taurus* (GCF_002263795.1) and *Rattus norvegicus* (GCF_000001895.5), respectively, have 1,637 and 377 annotated tRNA genes differentially distributed across potential anti-codons. Codon demand was determined from TAb weighted codon counts in the transcriptome. A consistent set of transcripts for codon usage calculations was constructed from across human transcripts which had data for both TAb and PAb available. For all other mammals, the orthologous transcripts were determined based on mammalian orthogroups from PaxDb (Wang et al., 2012).

The perturbability and resupply parameters were selected by optimizing the correlation between calculated TWnTE values and measured translation rates across all proteins in the Shwänhausser dataset (Schwanhäusser et al., 2011). The optimization was performed with R (R Core Team, 2013) using the neldermead package (Bihorel and Baudin, 2018) with initial estimates of 0.5 for both parameters. The perturbability parameter is normalized to an organism’s codon usage and tRNA availability, and the resupply parameter is based on basic diffusion. As only the Scwänhausser-based mouse dataset had translation rates, the TWnTE calculations for all other mammals used the same parameter values, under the necessary assumption that translation dynamics are consistent across the mammals tested.

Primate RNA-Seq data were acquired from the NonHuman Primate Reference Transcriptome Resource (Peng et al., 2015). Additional reads were downloaded via the Sequence Read Archive (Leinonen et al., 2011) for seven other transcriptomic studies (Brawand et al., 2011; Merkin et al., 2012; Fushan et al., 2015; Tang et al., 2017; Carelli et al., 2018; Valberg et al., 2018; Chen et al., 2019). The dataset assembled represents nine mammalian species: humans, chimpanzees, macaques, mice, rats, dogs, horses, cows, and pigs with data from six tissues: brain, heart, kidney, liver, lung, and muscle tissues. All RNA-Seq data were processed through the same pipeline to maximize consistency between datasets. Adapter removal, quality control, and read merging was performed using fastp (Chen et al., 2018) with quality windows of 4 bp, minimum quality thresholds of 20, a minimum read length of 30 bp, and merging any paired reads which overlapped by at least 20 bp with 80% similarity. TAb quantification was performed with Salmon (Patro et al., 2017) using reference transcriptomes acquired from RefSeq (O’Leary et al., 2016), and the validate mappings flag. As orphan reads were generated during quality control, quantification was performed separately for orphaned and paired reads for each sample before pooling the library-size-adjusted results together.

The number of genes or proteins for which data were acquired can be found in Supplementary table S1, Supplementary Material online. Total and LCR^+^ counts are broken down by data set, data type, species, and tissue.

Permutation testing was performed on the GTEx and PaxDB data by randomly shuffling the LCR status of proteins which had both TAb and PAB data. For each permutation, the first quartile, median, and third quartile of abundance was calculated for both the LCR^+^ and LCR^−^ groups. The difference between the values was recorded to establish null distributions for each quantile where LCR status is unrelated to abundance. The results of the permutation test of median abundance were verified with a Mann–Whitney *U*-test. The difference between each quantile was used rather than simply the difference between the median as it provides a better view of how LCRs are correlated with abundance across the wide distribution of abundances observed.

Permutation testing was also done as described above for several alternative conditions. To compare entropy-based and structure-based LCR identification, intrinsic disorder status was used as the permuted factor. To assess the effect of differential transcript stability, raw TAb was substituted with ischemia-time-adjusted TAb. To examine the effect of different classes of LCRs, separate permutation tests were performed which compared whether homo- or heteropolymer LCR^+^ proteins to LCR^−^ proteins. To assess if redundancy from protein isoforms was affected results, permutations were done using a subset of the data such that only one transcript-protein pair was used for each gene.

Logistic regression was used to assess the probability of a protein containing an LCR given the TAb and PAb, while accounting for differences in protein degradation rates, translation efficiency, and protein length. All regressors were log transformed to meet the assumptions of linearity, then all regressors were standardized to allow comparisons of their effects on LCR probability. The fold change in odds for a unit change in each regressor can be obtained by natural exponentiation of the estimated regression coefficients. When performing logistic regression on the mammalian RNA-Seq data, only TAb, and protein length were included as regressors. Regressions for all organisms were performed independently.

To evaluate the robustness of the analysis to the assumption that mouse translation parameters are applicable to other mammals, the logistic regression above was repeated for humans using standard nTE calculations, excluding translation efficiency from the model completely, and 25 pairs of parameter values evenly spread across the valid parameter space. The change to the estimated PAb and TAb coefficients was evaluated for qualitative changes to the conclusions.

## Supplementary Material

msac087_Supplementary_Data

## Data Availability

All data and software used in this work were acquired from the referenced, publicly available sources.
