# Supplementary material for: Low Complexity Regions in Mammalian Proteins are Associated with Low Protein Abundance and High Transcript Abundance
Source: Mol Biol Evol. 2022 Apr 28;39(5):msac087. doi: 10.1093/molbev/msac087 (PMC9070799; doi:10.1093/molbev/msac087)
Supplement: msac087_Supplementary_Data [file msac087_supplementary_data.pdf]

Low complexity regions in mammalian proteins are associated with low protein abundance and high transcript abundance

Zachery W Dickson and G Brian Golding

Department of Biology, McMaster University, Hamilton, Ontario, Canada

Supplementary Tables and Figures

Table 1: Number of genes/proteins with data by species and tissue

Dataset	Type	Species	Tissue	Total	LCR+
GTEx	TAb	Human	Aggregate	18067	4259 (23.6%)
			Brain	13903	3536 (25.4%)
			Colon	13981	3508 (25.1%)
			Esophagus	13694	3435 (25.1%)
			Heart	13223	3330 (25.2%)
			Kidney	13913	3437 (24.7%)
			Liver	12914	3180 (24.6%)
			Lung	14146	3524 (24.9%)
			Ovary	13582	3432 (25.3%)
			Pancreas	13291	3333 (25.1%)
			Prostate	14118	3532 (25.0%)
			Skin	13935	3491 (25.1%)
			Testis	15931	3839 (24.1%)
PaxDB	PAb	Human	Aggregate	20108	4246 (21.1%)
			Brain	8771	2437 (27.8%)
			Colon	7833	1944 (24.8%)
			Esophagus	6125	1434 (23.4%)
			Heart	10388	2675 (25.8%)
			Kidney	7427	1782 (24.0%)
			Liver	13341	3340 (25.0%)
			Ovary	11684	2986 (25.6%)
			Pancreas	9450	2437 (25.8%)
			Prostate	8992	2328 (25.9%)
			Skin	4267	950 (22.3%)
			Testis	12305	3158 (25.7%)
Schwanhäusser	TAb	Mouse	Fibroblast	3407	865 (25.4%)
	PAb			3407	865 (25.4%)
	k. Deg			4746	450 (9.5%)
	Trans. Eff.			3457	885 (25.6%)
Degredation	k. Deg	Human	Aggregate	8315	965 (11.6%)
RNA-Seq	TAb	Mouse	Brn/Hrt/Kdn/Lvr	70989	18094 (25.5%)
		Rat		55312	12915 (23.3%)
		Macaque		67926	18097 (26.6%)
		Chimpanzee		80690	20777 (25.7%)
		Human		108155	25233 (23.3%)
		Dog		58683	14908 (25.4%)
		Horse		60473	16543 (27.4%)
		Pig		63500	17571 (27.7%)
		Cow		63508	16756 (26.4%)

Table 2: Dataset specific estimates of wobble base-pairing selective constraints

Dataset	Species	AA	TG	GT	AC
GTEx	Human	1.000	0.3040	0.8406	1.370×10^{-2}
Schwanhäusser	Mouse	0.9945	1.190×10^{-2}	0.9678	1.470×10^{-2}
RNA-Seq	Mouse	1.000	0.5059	0.6665	1.049×10^{-3}
	Rat	1.000	0.2250	0.5793	9.999×10^{-7}
	Macaque	0.9991	0.6875	0.2960	2.600×10^{-4}
	Chimpanzee	0.9992	0.5311	0.2136	0.1583
	Human	0.9997	0.831	0.0629	0.1059
	Dog	1.000	0.6399	0.1149	4.700×10^{-4}
	Horse	0.9998	0.1196	0.1166	0.1417
	Pig	1.000	0.4881	0.3497	0.1288
	Cow	0.9999	3.528×10^{-5}	0.9999	0.9973

Table 3: Logistic regression using standardized GTEx and Schwänhausser data

Mammal	Proteins	LCR^+	Parameter	Transformation	$\bar{X}$	$SD_{\bar{X}}$	β	SE_β	Z value
Human	3107	903	(Intercept)	NA	NA	NA	-1.16	0.0628	-18.4
			Length	log2	8.78	1.060	-0.237	0.116	-2.040
			TAb	log2	0.638	2.87	0.244	0.0585	4.16
			PAb	log2	1.23	3.74	-0.212	0.0608	-3.49
			TWnTE	log2	-5.48	1.36	-0.910	0.112	-8.15
			k.deg	log	-3.83	0.852	0.147	0.0475	3.10
Mouse	2155	446	(Intercept)	NA	NA	NA	-1.38	0.0631	-21.8
			Length	log2	8.77	1.080	0.409	0.148	2.77
			TAb	log2	6.83	1.53	0.188	0.0801	2.35
			PAb	log2	3.81	3.24	-0.0843	0.0920	-0.916
			TWnTE	log2	-12.0	1.40	-0.449	0.200	-2.25
			k.deg	log	-4.20	1.030	0.115	0.0668	1.72

Table 4: Logistic regression using standardized mammalian RNA-Seq data

Mammal	Proteins	LCR^+	Parameter	Transformation	$\bar{X}$	$SD_{\bar{X}}$	β	SE_β	Z value
Chimp	73001	18614	(Intercept)	NA	NA	NA	-1.21	0.00948	-128
			Length	log2	9.020	1.13	-0.289	0.0291	-9.91
			TAb	log2	-0.564	2.44	0.0902	0.00914	9.87
			TWnTE	log2	-4.50	1.31	-1.080	0.0280	-38.7
Cow	58860	15584	(Intercept)	NA	NA	NA	-1.15	0.0105	-110
			Length	log2	8.98	1.090	0.561	0.0188	29.8
			TAb	log2	-0.0590	2.60	0.0901	0.0101	8.91
			TWnTE	log2	-14.9	1.14	-0.241	0.0211	-11.5
Dog	32599	8075	(Intercept)	NA	NA	NA	-1.27	0.0145	-87.1
			Length	log2	8.96	1.080	-0.381	0.0361	-10.6
			TAb	log2	0.457	2.74	0.0821	0.0141	5.81
			TWnTE	log2	-4.61	1.15	-1.080	0.0342	-31.7
Horse	52030	14389	(Intercept)	NA	NA	NA	-1.060	0.0107	-99.3
			Length	log2	9.040	1.11	-0.295	0.0289	-10.2
			TAb	log2	0.354	3.030	0.0477	0.0108	4.42
			TWnTE	log2	-4.55	1.29	-1.030	0.0282	-36.4
Human	88026	20201	(Intercept)	NA	NA	NA	-1.37	0.00908	-151
			Length	log2	8.94	1.090	-0.312	0.0291	-10.7
			TAb	log2	-0.252	2.64	0.115	0.00862	13.3
			TWnTE	log2	-5.89	1.35	-1.090	0.0282	-38.6
Macaque	63441	16578	(Intercept)	NA	NA	NA	-1.18	0.0101	-117
			Length	log2	8.99	1.12	-0.366	0.0312	-11.7
			TAb	log2	-0.0632	2.57	0.0830	0.00988	8.40
			TWnTE	log2	-4.58	1.39	-1.14	0.0302	-37.8
Mouse	54544	13644	(Intercept)	NA	NA	NA	-1.28	0.0118	-108
			Length	log2	8.96	1.10	0.330	0.0368	8.96
			TAb	log2	-0.419	3.060	0.0938	0.0107	8.75
			TWnTE	log2	-12.2	0.999	-0.569	0.0444	-12.8
Pig	55521	15144	(Intercept)	NA	NA	NA	-1.080	0.0104	-104
			Length	log2	9.030	1.070	-0.0985	0.0270	-3.65
			TAb	log2	-0.635	2.82	0.0418	0.0104	4.030
			TWnTE	log2	-4.22	1.20	-0.820	0.0260	-31.6
Rat	35562	8429	(Intercept)	NA	NA	NA	-1.37	0.0153	-90.1
			Length	log2	8.88	1.080	-0.280	0.0452	-6.20
			TAb	log2	-0.0723	3.060	0.0868	0.0136	6.40
			TWnTE	log2	-10.7	1.11	-1.15	0.0515	-22.3

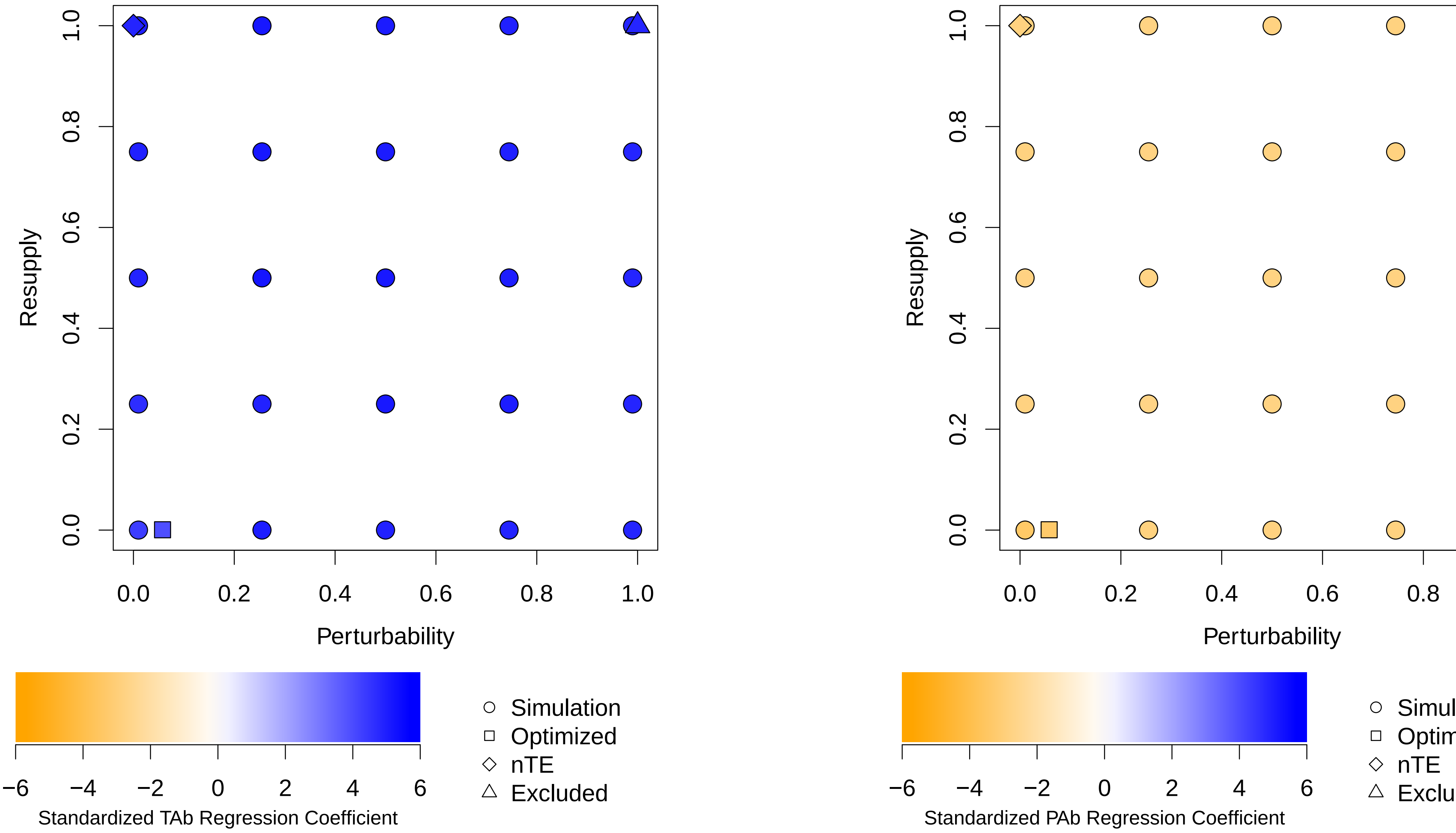

A **B**

Figure 1: Choice of translation parameters has no effect on the strength of the relationship between the presence of an LCR and TAb (A) and PAb (B). Colour indicates the z value of the regression coefficient when the indicated pair of translation parameters are used. Using standard nTE calculations is equivalent to a resupply of 1 and perturbability of 0. Excluding translation efficiency from the regression is positioned at (1,1) despite not having a true position in the parameter space.

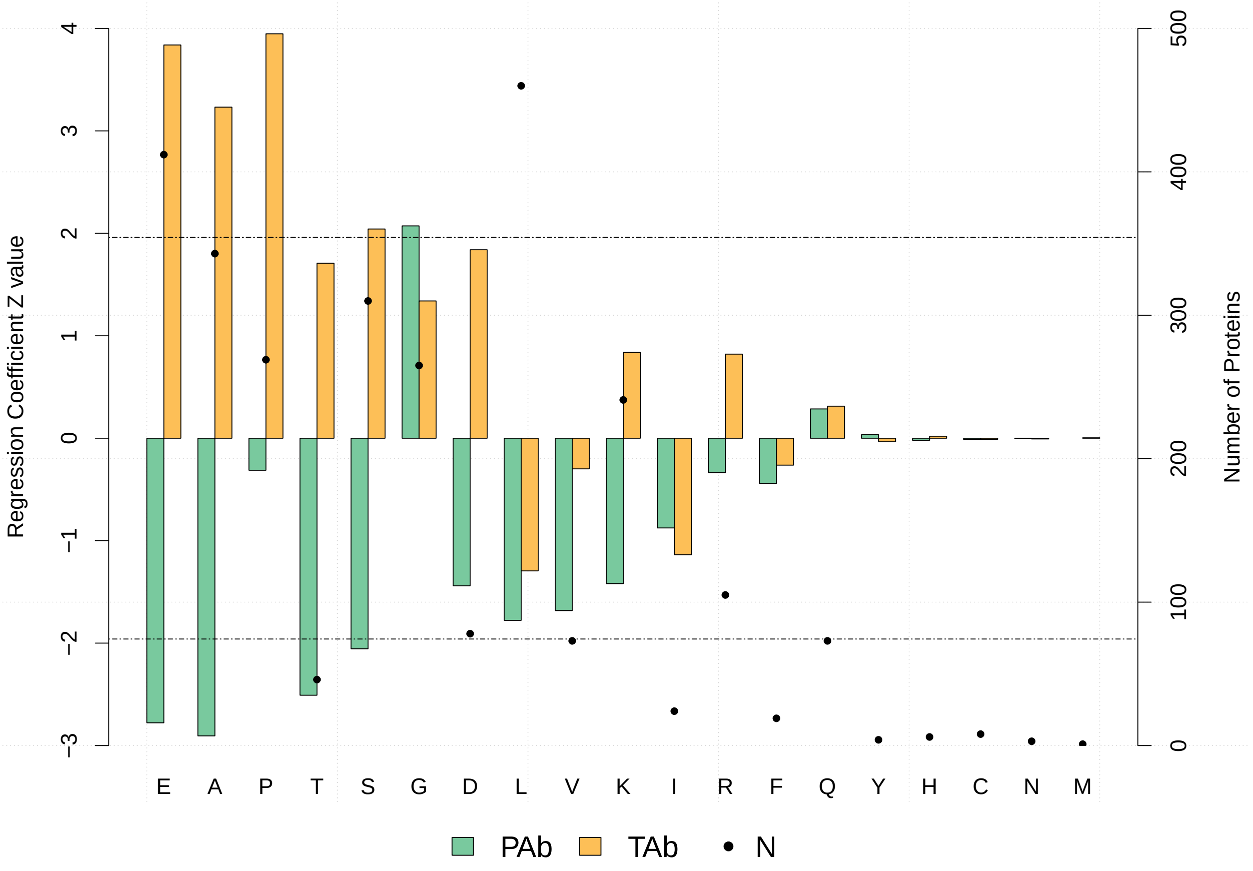

Figure 2: Aggregate LCR associations are also present for individial amino acids. Bars represent the z values of estimated logistic regression coefficients for the TAb and PAb with interaction terms for each amino acid. The amino acid for each protein is the amino acid which appears most often in the 15 AA windows of the protein sequence which have minimum entropy. The dotted line represents the z value corresponding to the 95% threshold. Individual points are the number of proteins with complete data which have the corresponding primary AA.
